# Patterns and risk factors for deaths from external causes in rural Malawi over 10 years: a prospective population-based study

**DOI:** 10.1186/s12889-015-2323-z

**Published:** 2015-10-09

**Authors:** Steady Chasimpha, Estelle McLean, Menard Chihana, Lackson Kachiwanda, Olivier Koole, Terence Tafatatha, Hazzie Mvula, Moffat Nyirenda, Amelia C. Crampin, Judith R. Glynn

**Affiliations:** Epidemiology and Population Health, London School of Hygiene & Tropical Medicine, Keppel St, London, WC1E 7HT UK; Malawi National Cancer Registry, Blantyre, Malawi; Karonga Prevention Study, Chilumba, Malawi

**Keywords:** Injury, Drowning, Suicide, Mortality rate, Alcohol, Malawi, Africa

## Abstract

**Background:**

Little is known about the pattern or risk factors for deaths from external causes in sub-Saharan Africa: there is a lack of reliable data, and public health priorities have been focussed on other causes. This study assessed the prevalence and risk factor for deaths from external causes in rural Malawi.

**Methods:**

We analysed data from 2002–2012 from the Karonga demographic surveillance site which covers ~35,000 people in rural northern Malawi. Verbal autopsies with clinician coding are used to assign cause of death. Repeated annual surveys capture data on socio-economic factors. Using Poisson regression models we calculated age, sex and cause-specific rates and rate ratios of external deaths. We used a nested case–control study, matched on age, sex and time period, to investigate risk factors for these deaths, using conditional logistic regression.

**Results:**

In 315,580 person years at risk (pyar) there were 2673 deaths, including 143 from external causes. The mortality rate from external causes was 47.1/100,000 pyar (95 % CI 32.5-68.2) among under-fives; 20.1/100,000 pyar (95 % CI 13.1–32.2) among 5–14 year olds; 46.3/100,000 pyar (95 % CI 35.8–59.9) among 15–44 year olds; and 98.7/100,000 pyar (95 % CI 71.8–135.7) among those aged ≥45 years. Drowning (including four deaths in people with epilepsy), road injury and suicide were the leading external causes. Adult males had the highest rates (100.7/100,000 pyar), compared to 21.8/100,000pyar in adult females, and the rate continued to increase with increasing age in men. Alcohol contributed to 21 deaths, all in adult males. Children had high rates of drowning (9.2/100,000 pyar, 95 % CI 5.5-15.6) but low rates of road injury (2.6/100,000 pyar, 95 % CI 1.0–7.0). Among 5–14 year olds, attending school was associated with fewer deaths from external causes than among those who had never attended school (adjusted OR 0.15, 95 % CI 0.08-0.81). Fishermen had increased risks of death from drowning and suicide compared to farmers.

**Discussion:**

In this population the rate of deaths from external causes was lowest at age 5–14 years. Adult males had the highest rate of death from external causes, 5 times the rate in adult females. Drowning, road injury and suicide were the leading causes of death; alcohol consumption contributed to more than one quarter of the deaths in men

**Conclusions:**

The high proportion of alcohol-related deaths in men, the predominance of drowning, deaths linked to uncontrolled epilepsy, and the possible protective effect of school attendance suggest areas for intervention.

## Background

The WHO estimates that more than 5.1 million deaths each year are attributable to injury and violence [1, 2], with 90 % of all such deaths occurring in low and middle income countries [3]. The importance of external causes of death is often overlooked in low income countries because of the high risk of death from other causes [3] and because there are few reliable data. Actual data from most low income countries are scarce or non-existent due to the lack of vital registration systems. A recent study from the INDEPTH network of demographic surveillance sites, including adults from Karonga, presented data on deaths from external causes by age and sex for different areas, providing the first population-level data for some countries [4]. That study used a probabilistic model (www.interva.net) to assign causes of death from verbal autopsy reports: this cannot use all available information, resulting in some misclassification, and the study did not look at risk factors for these deaths.

The WHO estimated mortality rates for deaths from external causes in 2012 were 73/100,000 population globally, 116/100,000 in the African region, and 98/100,000 in Malawi [2], but there are few direct data for Malawi or much of Africa. We have previously reported, from the demographic surveillance site in Karonga District, northern Malawi, that 5.2 % of deaths in adults in 2004–2009 were attributable to external causes (of a total crude adult mortality rate of 1105/100,000 person years at risk) [5]. In Chiradzulu district, Southern Malawi in 2008, 3/50 deaths were due to external causes [6]. Using police and hospital data, the mortality rate from road injury in Malawi was estimated at 19-21/100,000 person years at risk [7].

In this paper we present data on the patterns, rates and risk factors for deaths from external causes at all ages in a demographic surveillance site in northern Malawi from 2002–2012.

## Methods

### Setting

**Fig. 1:**
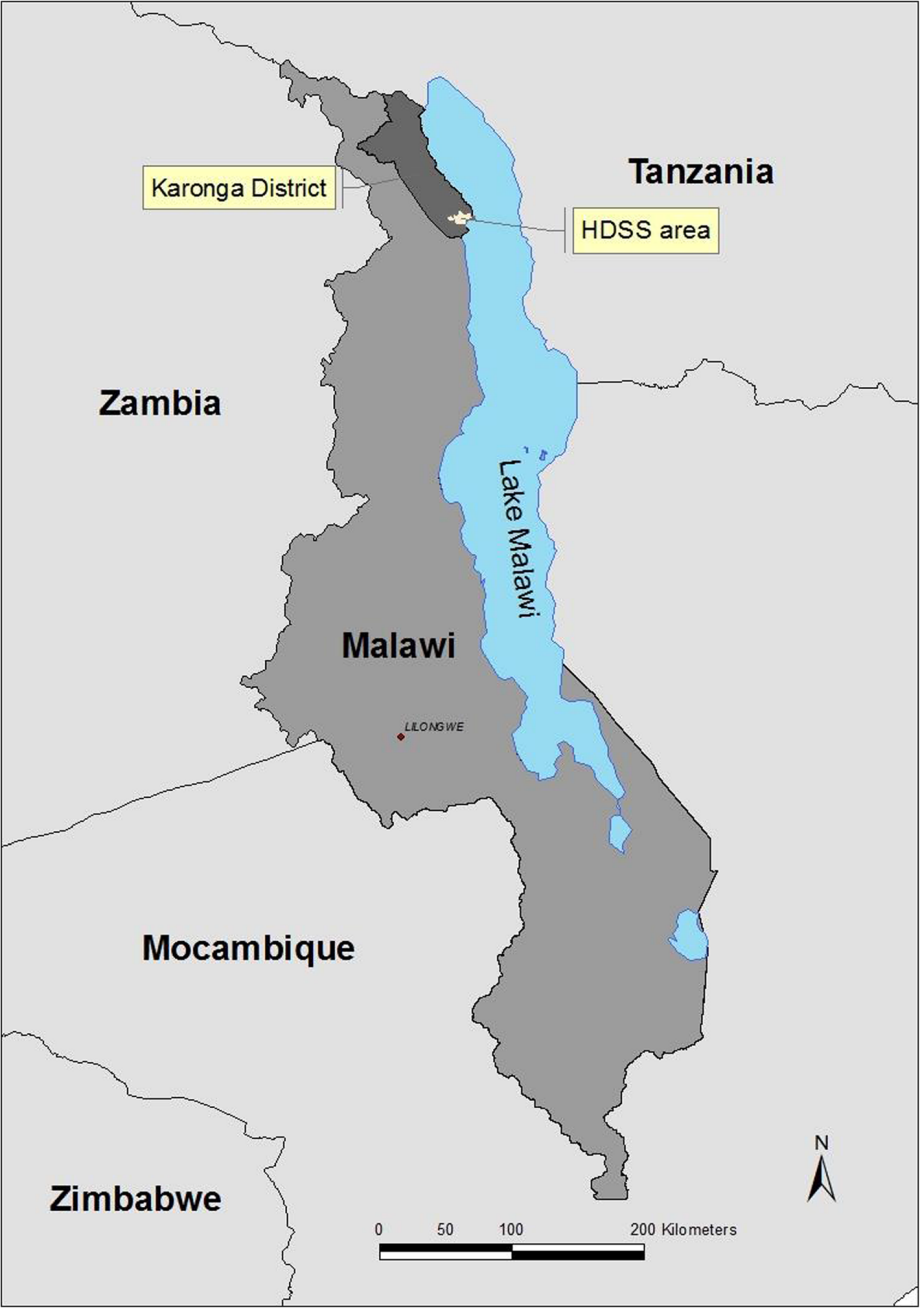
Study setting. The HDSS area is the health and demographic surveillance site

The Karonga Demographic Surveillance Site is situated in Karonga district in northern Malawi (Fig. 1). It was set up in August 2002 with a baseline census of all households in the area. Since then a continuous registration system based on trained village key informants records all vital events taking place in the area [8, 9]. The key informants report births and deaths monthly, and annual re-censuses are conducted to capture in and out-migration, check births and deaths, and collect socioeconomic information. HIV serosurveys were conducted from 2005, and data on HIV and on ART use are also available from linked studies in the same population. All HIV testing is done with informed consent, with results available to the participants. More than 35,000 people are registered in 8285 households in an area of about 135 km^2^. The population is predominantly rural, relying on subsistence farming, fishing (the site borders Lake Malawi) and small scale trading for their livelihood. About half the population live within one kilometre of the main tarmac road from Malawi to Tanzania, or of the tarmac spur road to the port.

### Cause of death

After a death report, a clinically trained interviewer visits the bereaved household, after a mourning period of 2–3 weeks, and fills a semi-structured verbal autopsy (VA) form. The forms are similar to the INDEPTH autopsy tool (2003) with some adaptations from the WHO questionnaire. Over the period of the study the VA tool in use has been modified to reflect changes in the WHO standardised questionnaire, however the questions regarding deaths from external cause have remained unchanged. Two clinicians (clinical officer or above) independently review completed VA forms and assign a cause or causes of death, with a third reviewer (MD-level equivalent or above) if the results are discordant [5, 10]. External causes of deaths are classified by intent: unintentional; intentional (suicide/assault); and unknown intent; and also by cause. To explore the role of alcohol in deaths from external causes, we extracted information about alcohol consumption from narratives on the VA forms. There was no direct question about alcohol consumption, so this information is available only if the respondent mentioned it. Alcohol-related deaths were defined as those assigned to direct alcohol poisoning or due to alcohol consumption of the person who died. It does not include injury or assaults due to alcohol consumption by others.

### Analysis

All analyses were performed using STATA version 13 (Stata Corp, College Station, TX, USA). Mortality rates and rate ratios were calculated overall and for specific causes of death using Poisson regression, looking at the patterns by age, sex and calendar period. Person years at risk (pyar) were defined from the date an individual was first seen in a census, date of birth if born in the demographic surveillance area since the start of the demographic surveillance, or the date of immigration into the demographic surveillance area. Follow up ended at the time of death whilst in the study area or at the time of out-migration from the area, or at the end of study period (31 December 2012). Multiple records and gaps existed for individuals who out-migrated and returned into the study area: only the time living in the study area was included in the calculation of person years at risk.

To investigate risk factors for deaths from external causes we used a nested case–control study. Cases were all those with deaths due to external causes. Controls were individually matched on year of birth of the case, sex and time period (using information close to the time of the cases’ death), using a random process to select up to 20 controls per case. This was done to ensure that the appropriate record was chosen for the controls for factors that change unpredictably over time, such as occupation or socio-economic status. Since such controls could go on to become a case (incidence density sampling) the odds ratios estimate the rate ratios that would be obtained from a cohort analysis [11]. Conditional logistic regression was used for the analysis. We fitted models in a step wise fashion starting with the variable with the strongest association, and keeping only variables with an effect on the outcome or confounding other factors in the final model. Multivariable analyses were done separately for children (<15 years), adult males and adult females as risk factors and causes of death are likely to differ. We also conducted separate analyses for the main external causes of death: road injury, drowning and suicide, and for deaths that were alcohol-related.

Ethics approval for the demographic surveillance and verbal autopsy studies was obtained from the National Health Sciences Research Committee in Malawi (#419) and Research Ethics Committee of the London School of Hygiene and Tropical Medicine.

## Results

Between August 2002 and December 2012, a total of 59,947 people were observed, representing 315,580 pyar. There were 2672 deaths, giving a crude mortality rate of 846.7/100,000 pyar. Cause of death was missing for 124 (3.4 %) deaths, and unknown for 89 (2.7 %) deaths. Overall, 143 (5.4 %) deaths were attributable to external causes giving a rate of 45.3/100,000 pyar (95 % CI 38.5-53.4). Among the external deaths 99 (69.2 %) were in males, rate 64.7/100,000 pyar (95 % CI 53.2-78.8) compared to 44 (30.8 %) in females, rate 27.1/100,000 pyar (95 % CI 20.1-36.4).

### Age-specific mortality rates and rate ratios

**Fig. 2:**
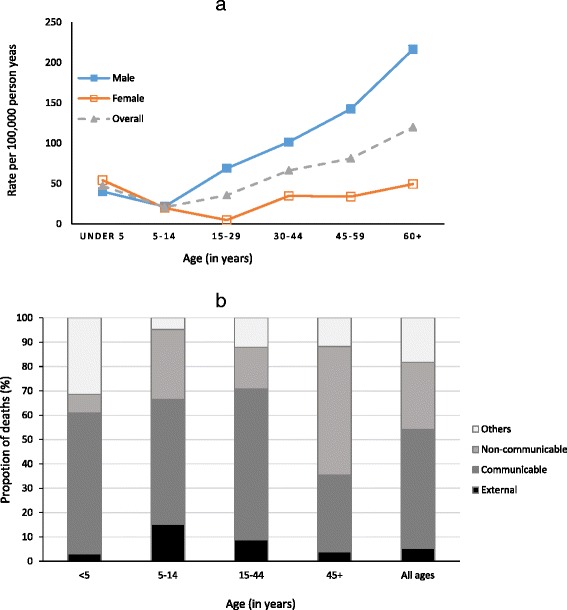
Deaths from external causes by age: **a** Rate of deaths from external causes by age and sex **b** Proportion of deaths due to different causes, by age

Figure 2 shows the mortality rate from external causes by age and sex, and the proportion of deaths due to external causes in different age groups. Rates are lowest at ages 5–14 years, although external causes account for a higher proportion of deaths in this age group than in others. After age 15 years the patterns for males and females diverge, with much higher rates in males. In males the rate increases steadily with age. In females the lowest rates were in age group 15–29 years, with no clear trend by age thereafter.

### Cause-specific mortality rates

Drowning, road injury and suicides were the leading external causes of death (Tables 1 and 2). Drowning accounted for 27 (18.9 %) unintentional deaths and two with unknown intent. Most of the unintentional drowning deaths were in children (14, of whom 8 were aged under 5) and adult males. The mortality rate from unintentional drowning in adult males was 15.7/100,000 pyar (95 % CI 8.9-27.7). More than half of all drowning deaths occurred at the lake and 4 were in those with known epilepsy (Table 2). There were 26 (18.2 %) road injury deaths, mostly in adult males (19), with only two in the under fives and two at age 5–14. Most road injury deaths involved pedestrians (51.9 %) compared to passengers (29.6 %), cyclists (11.1 %) and 7.4 % unknown. Suicides accounted for 22 (15.4 %) deaths, all in adults, and most (17) were in men.

**Table 1:** Rates of death from external causes in Karonga District Malawi, 2002–12

	All external causes		Road injury		Drowning		Suicide	
	Rate^a^	Rate ratio^c^	Rate	Rate ratio	Rate	Rate ratio	Rate	Rate ratio
	(95 % CI^b^)	(95 % CI)	(95 % CI)	(95 % CI)	(95 % CI)	(95 % CI)	(95 % CI)	(95 % CI)
Gender								
Male	64.7 (53.2–78.8)	Reference	12.4 (7.9–19.5)	Reference	13.1 (8.4–21.8)	Reference	11.1 (6.9–17.9)	Reference
Female	27.1 (20.1–36.4)	0.40 (0.28–0.57)	4.3 (2.1–9.0)	0.32 (0.14–0.77)	4.3 (2.1–9.0)	0.36 (0.16–0.81)	3.1 (1.3–7.4)	0.26 (0.09–0.70)
Age								
<5 years	47.1 (32.5–68.2)	0.99 (0.63–1.56)	3.4 (0.8–13.4)	0.27 (0.06–1.20)	13.4 (6.7–26.9)	1.83 (0.70–4.73)	0	
5–14	20.6 (13.1–32.2)	0.44 (0.26–0.73)	2.2 (0.5–8.7)	0.18 (0.04–0.77)	6.5 (2.9–14.5)	0.87 (0.31–2.46)	0	
15–44	46.8 (35.8–60.0)	Reference	12.0 (7.2–19.9)	Reference	7.2 (3.7–13.8)	Reference	13.6 (5.4–31.2)	Reference
45+	98.7 (71–135.7)	2.23 (1.48–3.36)	18.2 (8.7–38.2)	1.60 (0.65–3.92	10.4 (3.9–27.7)	1.52 (0.47–4.94)	13.0 (5.4–31.2)	1.01 (0.37–2.74)
Period								
2002/6	49.7 (38–64.8)	1.14 (0.81–1.60)	7.4 (3.7–14.7)	0.84 (0.35–1.92)	6.4 (3.1–13.5)	0.66 (0.28–1.56)	3.7 (1.4–9.8)	0.42 (0.14–1.22)
2007/12	43.0 (35.0–53.0)	Reference	8.7 (5.5–13.8)	Reference	9.7 (6.2–15.0)	Reference	8.7 (5.5–13.8)	Reference

^a^Rate expressed per 100,000 person-years-at-risk

^b^CI: Confidence Interval

^c^Rate ratio adjusted for age, sex and calendar period

**Table 2:** Detailed causes of death among deaths from external causes, Karonga District Malawi, 2002–12

Cause of death	Children (<15 years)		Adult Males (≥15 years)		Adult females (≥15 years)		Overall	
	Deaths	Rate^a^ (95 % CI^b^)	Deaths	Rate^a^ (95 % CI^b^)	Deaths	Rate^a^ (95 % CI^b^)	Deaths (%)	Rate^a^ (95 % CI^b^)
Unintentional								
Road injury	4	2.6 (1.0–7.0)	19	24.9 (15.9–40.0)	3	3.4 (1.1–1.7)	26 (18.2)	8.2 (5.6–12.1)
Drowning	14	9.2 (5.5–15.6)	12^d^	15.7 (8.9–27.7)	1^d^	1.1 (0.2–8.1)	27^e^(18.9)	8.6 (5.9–12.4)
Falls	3	2.0 (0.6–6.1)	2	2.6 (0.7–10.5)	5	5.7 (2.4–13.8)	10 (7.1)	3.2 (1.7–5.9)
Fire	3^f^	2.0 (0.6–6.1)	0		0		3^f^(2.1)	1.0 (0.3–2.9)
Alcohol poisoning	0		13	17.0 (9.9–29.3)	0		13 (9.1)	4.1 (2.4–7.1)
Other poisoning	4	2.6 (1.0–7.0)	0		0		4 (2.8)	1.3 (0.5–3.4)
Unspecifiable	1	0.7 (0.1–4.7)	0		0		1 (0.7)	0.3 (0.04–2.2)
Other^c^	14	9.2 (5.5–15.6)	3	3.9 (1.3–12.2)	2	2.3 (0.6–9.2)	19 (13.2)	6.0 (3.8–9.4)
Intentional								
Assault	1	0.7 (0.1–4.7)	8	10.5 (5.2–20.9)	1	1.1 (0.2–8.1)	10 (7.0)	3.2 (1.7–5.9)
Suicide–hanging	0		13	17.0 (9.9–29.3)	2	2.3 (0.6–8.1)	15 (10.5)	4.7 (2.9–7.9)
Suicide–poison	0		4	5.2 (2.0–13.9)	3	3.4 (1.1–10.7)	7 (4.9)	2.2 (1.1–4.7)
Unknown intent	3^g^	2.0 (0.6–6.1)	3	3.9 (1.3–12.2)	2	2.3 (0.6–9.2)	8^g^(5.6)	2.5 (1.3–5.1)
TOTAL	47	30.9 (23.3–41.2)	77	100.7 (80.6–123.0)	19	21.8 (13.9–34.1)	143	45.3 (38.5–53.4)

^a^Rate expressed per 100,000 person-years-at-risk

^b^CI: Confidence Interval

^c^Includes: 6 aspiration; 2 bee/wasp sting; 2 wall collapse; 1 crocodile attack; 1 lightning strike; 1 accidental child strangulation;1 Steven Johnson syndrome; 1 fractured cervical spine; 1 pail falling on them; 1 sudden death; 1 sepsis after knee puncture; 1 blow to head

^d^Two men and one woman were known to have epilepsy

^e^Includes: 15 in the lake; 5 in rivers/streams; 3 in wells, 1 in a drain; 1 in a basin

^f^Includes one child with epilepsy

^g^Includes two children found drowned, one of whom was known to have epilepsy

### Alcohol consumption related deaths

Overall 21 (15 %) external deaths involved alcohol consumption by the deceased, mortality rate 6.7/100,000pyar (95 % CI 4.3-10.2): 14 (66 %) were due to direct alcohol poisoning, four (19 %) were road injuries, while two (10 %) were assault and one (5 %) was drowned. All these deaths occurred in adult males, particularly older men (rate 23.2/100,000pyar (95 % CI 12.2–44.9) in men aged ≥45 years and 9.6/100,000 pyar (95 % CI 5.4–16.9) among 15–44 year-olds, calendar year-adjusted rate ratio 2.44; 95 % CI 1.07–5.80).

### Risk factors for deaths attributable to external causes

The 143 individuals with external deaths were compared to 2947 controls. There were 20 controls per case for 83 % of the cases, 95 % had at least 10 controls and all had at least two controls.

Table 3 shows that among children, only main household income source and individual schooling showed evidence of association with odds of death from external causes. Children from fishing households had three times the odds of external death compared to children in farming households (OR 3.07, 95 % CI; 1.03-9.10). This was due to drowning deaths: compared to farming households children from fishing households had greatly increased odds of death due to drowning (OR 21.9, 95 % CI 3.9–123.0). Children attending school had 85 % lower odds of external deaths compared to children with no schooling (OR 0.15, 95 % CI 0.08–0.81), excluding pre-school children. There was no association with schooling and any particular cause of death. The effects of schooling and main household income hardly changed when adjusted for each other.

**Table 3:** Risk factors for external deaths among children

Variable	Cases (%)	Control (%)	Odds ratio^a^	*P*-value^b^
	(*n* = 47)	(*n* = 1000)	(95 % CI)	
Sex (matched)				
Male	22 (46.8)	480 (48.0)		
Female	25 (53.2)	520 (52.0)		
Main household income				
Farming	11 (23.4)	411 (41.1)	Reference	
Fishing	5 (10.6)	60 (6.0)	3.07 (1.03–9.10	0.04
Business/other	17 (36.2)	385 (38.5)	1.74 (0.80–3.82)	0.17
Missing	14 (29.7)	144 (14.4)		
Dwelling construction				
Best	9 (19.2)	174 (17.4)	1.48 (0.59–3.76)	0.41
2	4 (8.5)	153 (15.3)	0.73 (0.22–2.38)	0.61
3	10 (21.3)	289 (28.9)	Reference	
Worst	11 (23.4)	236 (23.6)	1.36 (0.56–3.28)	0.49
Missing	13 (27.7)	148 (14.8)		
Not having enough food				
No	14 (29.8)	407 (40.7)	Reference	
Yes	6 (12.8)	139 (13.9)	1.27 (0.48–3.36)	0.64
Missing	27 (57.5)	454 (45.4)		
Father’s level of schooling				
None/primary	15 (31.9)	348 (34.8)	Reference	
Secondary	5 (10.6)	255 (25.5)	0.44 (0.16–1.22)	0.11
Missing	27 (57.5)	397 (39.7)		
Mother’s level of schooling				
None/primary	18 (38.3)	481 (48.1)	Reference	
Secondary	3 (6.4)	162 (16.2)	0.49 (0.14–1.72)	0.27
Missing	26 (55.3)	357 (35.7)		
Individual’s level of schooling				
None	12 (25.5)	235 (23.5)	Reference	
Any schooling	13 (27.7)	321 (32.1)	0.15 (0.03–0.81)	0.03
Pre-school^c^	20 (42.6)	423 (42.3)	2.34 (0.48–11.3)	0.29
Missing	2 (4.3)	21 (2.1)		

^a^Odds ratio with 95 % confidence interval controlled for age, sex and time period (by matching and conditional logistic regression)

^b^P-value: result of Wald test of association of each category

^c^Children under 5 years who had not yet attained school-going age

Among adult males, fishing – whether measured as individual occupation or as the main household income – was associated with death from external causes (Table 4). Fishermen had nearly three times the odds of external death compared to farmers (OR 2.92, 95 % CI 1.42–5.98). Individuals from fishing households had increased odds compared to those from farming households (OR 2.45, 95 % CI 1.17–5.14). Household income source was dropped from the multivariable model due to co-linearity. Adjusting for the other factors hardly changed the association with occupation.

**Table 4:** Risk factors for external deaths among adults

Variable	Adult males (≥15 years)				Adult females (≥15 years)			
	Cases (%)	Control (%)	Odds ratio^a^	*P*-value^b^	Cases (%)	Control (%)	Odds ratio	*P*-value
	(*n* = 77)	(*n* = 1584)	(95 % CI)		(*n* = 19)	(*n* = 342)	(95 % CI)	
Main household income								
Farming	30 (39.0)	676 (42.7)	Reference		7 (36.8)	149 (43.5)	Reference	
Fishing	11 (14.3)	100 (6.3)	2.45 (1.17–5.14)	0.02	1 (5.3)	29 (8.5)	0.74 (0.08–6.55)	0.08
Business/other	31 (40.3)	637 (4.2)	1.12 (0.66–1.88)	0.68	8 (42.1)	138 (40.4)	1.20 (0.41–3.51)	
Missing	5 (6.5)	171 (10.8)			3 (15.8)	26 (7.6)		
Occupation								
Farmer	46 (59.7)	993 (62.7)	Reference		9 (47.4)	255 (74.6)	Reference	
Fisherman	11 (14.3)	92 (5.8)	2.92 (1.42–5.98)	0.003				
Other	17 (22.1)	411 (26.0)	0.81 (0.44–1.52)	0.52	8 (42.1)	63 (18.4)	4.04 (1.22–13.4)	0.02
Missing	3 (3.9)	88 (5.6)			2 (10.5)	24 (7.0)		
Dwelling construction								
Best	13 (16.9)	340 (21.5)	0.73 (0.36–1.46)	0.37	6 (31.6)	77 (22.5)	Reference	
2	13 (16.9)	223 (14.1)	1.10 (0.53–2.27)	0.80	2 (10.5)	59 (17.3)	0.48 (0.09–2.51)	0.39
3	23 (29.9)	466 (29.4)	Reference		4 (21.1)	106 (31.0)	0.59 (0.16–2.17)	0.43
Worst	21 (27.3)	379 (23.9)	1.08 (0.58–2.00)	0.81	5 (26.3)	71 (20.8)	0.56 (0.26–3.96)	0.84
Missing	7 (9.1)	176 (11.1)			2 (10.5)	29 (8.4)		
Not having enough food								
No	28 (36.4)	792 (50.0)	Reference		6 (31.6)	165 (48.3)	Reference	
Yes	14 (18.2)	259 (16.4)	1.46 (0.75–2.86)	0.26	3 (15.8)	54 (15.8)	1.93 (0.44–8.48)	0.87
Missing	35 (45.5)	533 (33.6)			10 (52.6)	123 (36.0)		
Individual’s level of schooling								
None/primary^c^	47 (61.0))	924 (58.3)	Reference		16 (84.2)	275 (80.4)	Reference	
Secondary	29 (37.7)	632 (39.9)	0.91 (0.55–1.49)	0.70	3 (15.8)	60 (17.5)	0.98 (0.25–3.96)	0.87
Missing	1 (1.3)	28 (1.8)			0	7 (2.1)		
HIV status								
Negative	26 (33.8)	995 (62.8)	Reference		6 (31.6)	228 (66.7)		
Positive	5 (6.5)	89 (5.6)	2.05 (0.72–5.79)	0.18	0	20 (5.8)		
Missing	46 (59.7)	500 (31.6)			13 (68.4)	94 (27.5)		

^a^Odds ratio with 95 % confidence interval controlled for age, sex and time period (by matching and conditional logistic regression)

^b^ *P*-value: result of Wald test of association of each category

^c^There were few cases with no schooling hence combined with primary education group

The increased risk of death among fisherman was seen both for drowning (OR 8.9, 95 % CI 2.0–39.8) and suicides (OR 6.1, 95 % CI 1.5–24.0). HIV positivity was weakly associated with an increased risk of external death overall (OR 2.1. 95 % CI 0.7–5.8) but strongly with alcohol-related deaths: OR 32.9, 95 % CI 3.5–311.6. There was no association between HIV status and suicide (no reported suicides among those known to be HIV positive) but HIV status was unknown for many.

Among adult females, those in occupations other than farming, which include small traders, salaried occupations and piece work, had a higher odds of death from external causes (OR 4.04, 95 % CI 1.22–13.4). No other associations were found, but there were only 19 external deaths in women.

## Discussion

This study highlights the very different rates of death from external causes by age and sex, and identifies some associated risk factors. The overall rate was lower than estimated by WHO for Malawi [2], but in line with the rate for other, mostly rural, demographic surveillance sites in Africa [4]. Most deaths occurred among adult males (100.7/100,000 pyar vs 21.8/100,000 pyar in adult females and 30.9/100,000 pyar in children). Although the proportion of deaths due to external causes was highest among 5–14 year olds, this reflected low rates of deaths from other causes, and this age group actually had the lowest mortality rate for external causes. After age 15, the rates rose steadily with age in men, but remained low in women. The three predominant causes of death: drowning, road injury and suicide were all more common in men than in women.

Among adult males 27 % (21/77) of deaths were related to alcohol consumption in the deceased, compared to none in women; but this only partially explains the higher rates of external deaths in men. The increased rate of alcohol-related deaths in HIV positive men could be a response to their HIV status, but could be a marker of lifestyle factors common to alcohol use and sexual risk taking. The role of alcohol was probably underestimated: it may not always be known or divulged by the informant and we could not assess the role of alcohol consumption amongst perpetrators of violence or drivers of cars that killed others. The high rates of death from direct alcohol poisoning may be related to the availability of locally distilled alcohol, which typically contains many toxic impurities. In recent years there has been a rapid increase in sales and consumption of strong alcohol sold very cheaply in 100 ml plastic sachets from unlicensed retailers [12]. This is reported sometimes to contain non-food alcohol [13]. Malawi has developed a model National Alcohol Policy [14] and there are plans for a ban on the sachets [15]; the impact on alcohol related deaths may be assessed in the future.

Children and adult males had high rates of drowning. Most of these deaths were in the lake, which is used for washing clothes, bathing and swimming as well as fishing. Four drowning deaths (including one with unknown intent) were probably related to epilepsy. Many of the interventions against drowning that have been used successfully in high income settings are inappropriate for rural and low income settings with large bodies of water [16]. The WHO produced their first exclusive report on drowning in 2014 [17]. Key risk factors identified included lack of barriers, lack of close supervision for young children, poor swimming skills and a lack of awareness of water dangers. The report also emphasised a link with alcohol. In Karonga barriers would be impossible – the district runs along the lakeshore (Fig. 1). Improved supervision of children may help, and fits with the observation that attending school was protective against external deaths (OR 0.15, 95 % CI 0.08–0.81). This may be because it reduces the time at risk (for example playing in the lake) rather than, or as well as any direct effect of education. Greater supervision of preschool children might reduce their risk.

Children had surprisingly low rates of death from road injury, given that a main road with fast traffic passes through the area, and there is a truck stop, with minimal provision for pedestrians and no lighting. Deaths from burns were low (rate 1.0 /100,000 pyar, 95 % CI 0.3–2.9), about a third of the rate in other places such as South Africa (3.8/100,000), [18] with no fatal burns in adults, and the only fatal case in a child over 5 was related to an epileptic fit. Elsewhere some fatal burns result from self-immolation, a method of suicide not seen in this population. The low rate of fatal burns in children is surprising given that small children are frequently close to cooking pots and fires in external kitchens in this setting.

The findings reiterate the importance of age and sex as predictors of injury-related deaths, as elsewhere [19–21]. The high rate of drowning deaths among children, and the predominance of deaths from road injury and suicide have also been reported worldwide [4, 22–25]. In our setting, successful suicide-by-hanging prevailed over suicide-by-poison, although anecdotally there are more suicide attempts through poisoning. In other parts of Malawi, poison has been shown to be a common method of suicide [26]. Suicide and attempted suicide remain illegal in Malawi [27] and prosecutions of survivors can occur, so it is possible that some informants concealed their suspicions of intent. There were few assault related deaths (7 %: 3.2/100,000 pyar, 95 % CI 1.7–5.9): much higher rates have been reported in South Africa and Kenya [4].

This is a large population based cohort study with long follow-up, a rigorous prospective continuous registration system, and clinician coding of verbal autopsy reports. These should be reliable for assigning deaths as due to external cause and should be more accurate than methods based on hospital or police reports. Although the study area is small, it is reasonably typical of many rural areas in Africa. The lakeshore setting clearly contributed to the high rates of drowning.

## Conclusions

This study has highlighted large sex differences in deaths from external causes and shows that although the proportion of deaths due to external causes is highest in children and young adults, the rate of deaths from external causes is relatively low in these groups. Alcohol accounted for more than a quarter of deaths in men. This, together with the predominance of drowning, the deaths in those with uncontrolled epilepsy, and the possible protective effect of school attendance, give pointers to prevention strategies [25].

## Abbreviations

CI: Confidence interval
OR: Odds ratio
Pyar: Person years at risk
VA: Verbal autopsy
WHO: World Health Organisation
